# Goal-directed therapeutic exercise for paediatric posterior fossa brain tumour survivors: a qualitative analysis of experiences

**DOI:** 10.1007/s00520-024-08327-3

**Published:** 2024-01-22

**Authors:** Brooke E. Kohler, Emmah Baque, Carolina X. Sandler, Taryn Jones, Caroline O. Terranova, Denise S. K. Brookes, Timothy Hassall, Natalie K. Bradford, Stewart G. Trost

**Affiliations:** 1 Faculty of Health at the Queensland Centre for Children’s Health Research, Queensland University of Technology (QUT) Brisbane, Brisbane, Australia; 2 School of Health Sciences and Social Work, Griffith University, Nathan, Australia; 3 Sport and Exercise Science, School of Health Sciences, Western Sydney University, Sydney, Australia; 4 UNSW Fatigue Research Program, Kirby Institute, University of New South Wales, Sydney, Australia; 5 Menzies Health Institute Queensland, Griffith University, Brisbane, Australia; 6 Children’s Health Queensland, Brisbane, Australia; 7 Cancer and Palliative Care Outcomes Centre, at Centre for Children’s Health Research, Queensland University of Technology, South Brisbane, Australia; 8 School of Human Movement and Nutrition Sciences, University of Queensland, Brisbane, Australia

**Keywords:** Children, Oncology, Brain tumour, Physical activity, Survivorship, Interview

## Abstract

**Purpose:**

To explore child and parent experiences of a 12-week goal-directed therapeutic exercise intervention in paediatric posterior fossa brain tumours survivors and to identify features of the program that influenced program adherence and acceptability.

**Methods:**

Eleven interviews were conducted; five parent-child dyads (mothers = 83%) and one parent only (mean child age = 10.6 ± 3.0 years; 83% male). Posterior fossa brain tumour survivors, who participated in a weekly goal-directed exercise program for 12 weeks, completed semi-structured interviews to discuss their experience of the program. An inductive content analysis was undertaken. Interviews were transcribed, imported into NVivo and independently coded by two reviewers. Code and content categories were iteratively discussed and refined.

**Results:**

Five content categories were generated: (1) perceived improvements, (2) program logistics, (3) activity selection, (4) connection with the therapist and (5) options for technology. All participants valued the tailored exercise program and described improvements in movement competence. Children and their parents discussed preferring home- and community-based locations and favoured face-to-face delivery. Occasionally, parents reported difficulty completing the home program due to low child motivation or family time restrictions. Multiple families suggested an interactive digital application would be an effective delivery channel for the supplemental home-based program.

**Conclusion:**

A goal-directed exercise program delivered at home and in community-based locations was considered valuable and helpful for improving movement competence in paediatric survivors of posterior fossa brain tumour.

**Trial registration:**

ACTRN12619000841178 June 12, 2019

## Background

Brain tumours account for 25% of all childhood cancer diagnoses, with incidence rates increasing over the past three decades [1, 2]. Improvements in medical interventions have led to improved survival rates, meaning more children than ever are living with the consequences of a brain tumour and its treatment [1, 2]. The most common tumour location is in the posterior fossa of the skull, effecting the cerebellum and adjacent areas [3]. Children surviving posterior fossa brain tumours (PFBT) experience a myriad of disease- and treatment-related consequences, including poor cardiorespiratory fitness, reduced muscle strength, impaired balance and coordination, impaired cognitive function and lower quality of life, even compared to other cancer types (e.g., acute lymphoblastic leukaemia) [4–8].

Difficulties with physical and cognitive skills suggest survivors are less likely to keep up with peers at school, perform age-appropriate activities of daily living and be involved in high-intensity physical activities [5, 9, 10]. Attenuated by reduced physical performance, childhood brain tumour survivors report lower levels of physical activity, further predisposing survivors to disabling chronic health conditions later in life [11, 12]. Survivors of childhood cancer are 3.3 times more likely than siblings to be diagnosed with a chronic health condition (e.g., atherosclerosis, diabetes) in adulthood [12]. Cancer in childhood is further complicated by the disruption to critical periods of growth and development, meaning the implications to cognitive and physical development may be significant and lifelong. Interventions aimed at ameliorating the negative effects of the tumour and its treatment are needed to inform children, families and clinicians of how to maximise quality survivorship and effectively manage the short- and long-term complications.

Regular participation in physical activity is both preventative and therapeutic for managing chronic health conditions [13]. To date, physical activity interventions in childhood cancer survivors have largely focused on those with blood cancers (e.g., acute lymphoblastic leukaemia) during treatment [14–16]. Physical activity of sufficient frequency, intensity and duration provide benefits to survivor’s cardiorespiratory fitness, muscle strength, cognitive functioning and fatigue [14–16]. In the last decade, there is emerging evidence that exercise is safe, feasible and potentially effective to improve the health and well-being of childhood brain tumour survivors [17, 18]. Intervention types vary greatly, with most studies implementing adult-like exercise modalities (e.g., cycle ergometers, strength training), with a distinct lack of family-centred or goal-directed intervention approaches [17]. Goal-directed therapeutic exercise is highly individualised, it promotes attention in and commitment to therapy, encourages autonomy and considers the patient’s interests, preferences, current level of function and environmental factors [19]. Evidence supports the effectiveness of family-centred, goal-directed interventions to improve motor activities, habitual physical activity and quality of life in other paediatric patient groups (e.g., cerebral palsy, muscular dystrophy, intellectual disability); however, there is a paucity of evidence in childhood cancer survivors [19–21].

The Physical ACTivity in Survivorship (PACTS) trial is an ongoing single-centre randomised controlled trial (RCT) designed to address the knowledge gap in relation to the effectiveness of goal-directed exercise programs for childhood cancer survivors [22]. The primary aim of PACTS is to evaluate the effects of a 12-week goal-directed therapeutic exercise intervention on physical activity-related goal attainment and cardiorespiratory fitness in children aged 5 to 17 years previously diagnosed with a PFBT. Participants randomised to the intervention program received personalised exercise therapy, catered for their goals, age, activity preferences, current level of function, environment and presence of comorbidities. This is a novel approach in paediatric oncology; therefore, its acceptability among children and parents is largely unknown. The aim of this study was to explore children and parents’ experiences of participation in the PACTS goal-directed exercise intervention and to identify features of the program that helped or hindered program adherence and acceptability.

## Methods

### PACTS RCT

This qualitative study was undertaken as part of the PACTS study [22]. The trial was prospectively registered with the Australian New Zealand Clinical Trials Registry (ACTRN12619000841178). Ethical approval was granted from the Human Research Ethics Committee of Children’s Health Queensland (HREC/19/QCHQ/50464) and the Queensland University of Technology (1900000696). To be included in the study, participants were: aged between 5 and 17 years; have had a diagnosis of a PFBT; undergone surgery for a PFBT a minimum of 12 months earlier, with no maximum time since surgery providing age eligibility was met; have completed radiation and/or chemotherapy in the previous 6 months; have no evidence of progressive disease and were deemed medically able to complete an exercise program, as determined by the treating oncologist. Participants were excluded if they had insufficient literacy skills or were unable to travel to the research facility to complete study assessments. The assessment of literacy skills and suitability for the study was determined by the study oncologist and neuro-oncology research nurse at the time of recruitment. In total, 50 families were identified in the Paediatric Oncology Unit patient database and contacted. Twenty-six families declined study involvement for the following reasons: commitment too great (*n* = 9), child too busy (*n* = 5), distance too great (*n* = 4), parent/s too busy (*n* = 3) or other reasons (*n* = 5). Five families were not able to be contacted, due to either a disconnected number or no response. Eighteen children were randomised to the intervention (*n* = 9) or the control group (*n* = 9).

Participants randomised to the intervention group received weekly individualised, goal-directed exercise therapy delivered face-to-face for 12 weeks by a qualified physiotherapist. Exercise sessions were 30 to 60 min in duration at a location of convenience to the family. Location options included the research facility, the participant’s home and/or public parks and pool facilities. The intervention adhered to accepted principles for exercise prescription for children and adolescents, with cardiorespiratory, strength and goal-directed task-specific components prescribed each session. At least half of each exercise sessions was dedicated to cardiorespiratory focused activities, with a heart rate ≥ 140 bpm. Goals selected for the intervention were related to physical activity and included goals related to sports, exercise and/or recreation. The individualised exercise program was structured according to the participant’s age, interests, current level of function and presence of treatment-related and disease-related side effects. To complement the in-person sessions, participants completed a home program three times per week, with each session up to 30 min in duration. The home-based program replicated the essential elements of the in-person therapist supported sessions and utilised resources available at home (e.g., supervision, equipment). Before initiating the program, the treating therapist completed a home visit to determine what space, equipment and resources were available. Child participants and their parents were given detailed printed instructions and pictures of the home program and a logbook to track completed activities. To promote adherence, peers and siblings were encouraged to participate in the home activities. Participants randomised to the control group received care as usual. Usual care included any therapy and visits to their treating medical practitioner.

### Participant goals

Participant goals were set in collaboration with the therapist during the participant’s baseline assessment. Goals related to physical activity performance and were all activity- or participation-focused. Examples of participant goals are listed in Table 1.

**Table 1:** Examples of participant goals

Goal type	Goals: to be able to …
Activity-focused	1. Ride an adapted trike around the cul-de-sac at home, for greater than 5 min before requiring a rest. 2. Dribble a basketball using my dominant hand the length of the basketball court without losing control of the ball. 3. Perform a two-foot jump with the skipping rope ten times in a row at school. 4. Run 100 m without stopping. 5. Throw a tennis ball and catch it with a partner, standing 3 m apart, with >75% accuracy.
Participation-focused	1. Keep up with my friends during 20–30 min of lunchtime play. 2. Play a game of soccer with my siblings in backyard, for greater than 5 min before requiring a rest. 3. Participate in >50% of activities during a Physical Education class at school. 4. Play a 20-min game of tennis with a family member at the local court once a fortnight. 5. Play a whole game of interschool sport this term.

### Participants and recruitment

Children and their parents who completed the PACTS exercise intervention were invited to participate in semi-structured interviews to discuss their experiences participating in the exercise program. Families were contacted by phone following the completion of the program by a member of the research team not involved in the delivery of the program. Information sheets regarding the interview component of the PACTS study were provided to each of the participants and consent was provided. Full written and informed consent was obtained from legal parents/guardians of all participants, verbal assent for children aged 7 to 13 years and verbal and written assent for children aged 14 years or older at study enrollment. Parent and child verbal consent was obtained at the beginning of each interview.

### Data collection

The child and parent interview guides were developed collaboratively by the investigators and based upon the essential elements of the PACTS intervention program (see Online Resource 1). The interview guides were piloted with a child and parent not involved in the study, and responses were discussed with investigators (BK, EB, CS, NB, SGT). Interviews were conducted via videoconference from May to July 2022 by a health professional familiar with, but not directly involved with the PACTS study (TJ). Data collection continued until all eligible families had been approached. Recently, research suggests that traditional data saturation may not be possible or appropriate for qualitative research of some novel or exploratory studies, rather a discovery of new insights is suitable for data collection [23]. Each interview was conducted in a conversational style, using open-ended questions, probing and clarifying questions and with appropriate changes in language and tone to suit the needs of each child [24]. While individual responses were encouraged, all perspectives of the PACTS program were welcomed including child- and parent-only responses or parent-assisted responses. Parents were offered to participate in their interview first, followed by the child. Interview guides were used separately. Interviews were audio recorded on the videoconferencing platform, stored on a secured server and transcribed verbatim by a certified transcription service with strict confidentiality and privacy practices (ISO 9001 and ISO 27001 certification). Each interview lasted between 10 and 45 min.

### Data analysis

An inductive content analysis was undertaken. Inductive content analyses are useful for novel topics, where data-driven analysis derives meaning and brings immediate relevance to its readers [25–27]. Transcripts were imported into NVivo (QSR International Pty Ltd., Burlington, USA) for data coding and analysis. All transcripts were read and cross-checked against the original recording by one investigator (BK). All investigators met to establish a preliminary coding structure informed by the interview guides. Following this, two investigators independently coded transcripts from two parents (BK and CS) and two child participants (BK and EB) then met again to compare the resultant coding. One coder (BK) was involved in the delivery of the intervention; the other coders (CS and EB) were familiar with the project but had no contact with participants. Interrater reliability was calculated through Kappa coefficient using NVivo, where coded data below a Kappa coefficient of 0.75 were discussed as a group (BK, CS, EB and NB) and consensus was reached [28]. The remaining transcripts were then coded by one investigator (BK) according to the agreed coding structure. Codes were then grouped into content categories. For this process, transcripts were re-read, and codes and content categories were iteratively discussed by members of the research team and refined as needed. Investigators moved between the data and analyses to ensure findings were unbiased with a distanced analysis [25–27]. All investigators agreed on the final structure and content categories. Content categories were then summarised narratively and are presented here with supporting quotations. The results were organised and presented in accordance with the Consolidated Criteria for Reporting Qualitative Research (COREQ) checklist [29].

## Results

### Participant characteristics

All eight child-parent dyads who participated in the PACTS intervention were approached. Of these, five child and six parent interviews were conducted. Two families did not respond to attempts to contact, and one child was unwell at the time of the interview. Table 2 details child and parent characteristics. Children were mostly male (83%), aged 10.6 ± 3.0 years and were 4.2 ± 1.3 years post-treatment at study baseline. Interviews were conducted 8.1 ± 4.4 months after intervention completion. Most parent interviews were conducted with mothers (83%), who had university-level qualifications (83%) and were all middle- to high-income earning families. The characteristics of the included sample did not substantially differ, largely reflecting the characteristics of the full intervention group of the PACTS study.

**Table 2:** Participant characteristics from the PACTS study interviews

Characteristics	Included participants (*n* = 6)	All intervention participants (*n* = 8)
Child	Mean ± SD (range)	
Age at study baseline (years)	10.6 ± 3.0 (5.4 – 14.7)	10.7 ± 3.9 (5.1 – 16.7)
Age at tumour diagnosis (years)	5.6 ± 2.3 (1.7 – 8)	5.9 ± 2.7 (1.7 – 10.1)
Time since end of treatment to study baseline (years)	4.2 ± 1.3 (2.3 – 5.1)	4.6 ± 2.4 (2.3 – 9.6)
Time since completion of intervention to interview (months)	8.1 ± 4.4 (1.8 – 14.1)	
	*n* (%)	
Male	5 (83%)	6 (75%)
Aboriginal or Torres Strait Islander	0 (0%)	0 (0%)
Tumour type		
Cerebellar astrocytoma	4 (67%)	5 (63%)
Medulloblastoma	1 (17%)	1 (13%)
Brain stem glioma	1 (17%)	1 (13%)
Ependymoma	0 (0%)	1 (13%)
Treatments received		
Surgery only	4 (67%)	5 (63%)
Surgery, chemotherapy and radiation therapy	2 (33%)	3 (38%)
Intervention location		
Home	2 (33%)	3 (38%)
Research facility	1 (17%)	2 (25%)
Mixed home and research facility	3 (50%)	3 (38%)
Adherence, average percentage of planned sessions completed		
In-person (12 sessions scheduled)	94.7%	95.8%
Home program (three sessions per week scheduled)	61.5%	67.5%
Parent	*n* (%)	
Relationship to the child		
Mother	5 (83%)	
Father	1 (17%)	
Parent education level		
Postgraduate university degree	2 (33%)	2 (25%)
Undergraduate university degree	3 (50%)	4 (50%)
Grade 12	1 (17%)	2 (25%)
Family structure		
One-parent household	1 (17%)	1 (13%)
Two-parent household	5 (83%)	6 (75%)
Other (e.g., grandparents)	0 (0%)	1 (13%)
Annual household income		
≥ $198,796 per year	2 (33%)	3 (38%)
$150,592 – $198,744 per year	1 (17%)	1 (13%)
$119,912 – $150,540 per year	2 (33%)	2 (25%)
$81,380 – $99,892 per year	1 (17%)	2 (25%)

### Content categories

The following five content categories were identified from the analysis: (1) perceived improvements, (2) program duration, frequency, location and format (program logistics), (3) activity selection, (4) connection with the therapist and (5) options for technology. Overarching all findings was a general positive assessment of the PACTS program; all parent and child participants expressed liking the program. The in-person sessions with the therapist were perceived as helpful and enjoyable.

I thought it was good. I thought it was quite interesting from our side of things. Everybody was really lovely. Very accommodating. Very nice. Kind to Ch04. It was well organised. So, it was a good experience. (Ch04’s mother, Ch04 age 10, cerebellar astrocytoma, mixed home and research facility sessions).

I thought the overall program was excellent. I really did. I thought it was great. Once we got started and got into it and yeah, right up until the end … He loved it. He thought it was great. He thought it was fun. You know, it was engaging (Ch05’s mother, Ch05 age 11, medulloblastoma, research facility sessions).

#### Perceived improvements

All parents perceived positive changes in their child’s movement competence. Greater confidence and acquiring new skills were the most frequently reported changes. The new skills were primarily aligned with the participant’s goals, although some reported improvements in other areas (e.g., running faster). There was a notion that the exercise program was an opportunity to ‘catch up’ on skills that were lost or delayed during the intensive phase of medical treatment. Furthermore, some parents spoke about the benefit of goal setting and how receiving regular feedback assisted motivation.

Well immediately and during the six weeks of the exercise sessions, we noticed a huge improvement in her coordination, her balance, her energy levels. Her skills in terms of - we really saw her skills improve over the week in terms of her ability to run and jump and move quickly (Ch04’s mother, age 10, cerebellar astrocytoma, mixed home and research facility sessions).

He now knows and we know that he’s capable of more than he thought, especially with distance, dragging him around somewhere, although he’d get tired, he knows he’s capable of it (Ch01’s mother, age 14, brain stem glioma, home sessions).

… you know, an outcome sometimes of being hospitalised and all of that is they lose confidence and they tend to kind of withdraw a bit … So they kind of feel a little bit isolated. So, I guess it was just him seeing that yes, we're going to learn to ride a bike. We're going to do those things that all the other kids have done. (Ch06’s mother, age 11, cerebellar astrocytoma, home sessions).

Just that constant, I guess awareness that the effort you are putting in is paying off, kind of helps encourage them to keep going and put in the effort, which he did. (Ch01’s mother, age 14, brain stem glioma, home sessions).

Child participants also reported improvements in skills and general physical function attributes.

I guess I’ve been a little more confident in my catching ability … Someone chucked a pen at me in class and I caught it, and I was like, oh, yeah (Ch01, age 14, brain stem glioma, home sessions).

Well, I think I'm faster and stronger (Ch02, age 10, cerebellar astrocytoma, home sessions).

Additionally, some parents felt the exercise program was the prompt they needed to be more active as a family.

Because it was something that I knew I had to do as a parent, it was kind of like I've got to get this done, I've got to get this done, I couldn’t say forget it then. Just go inside and play. Do you know what I mean? I felt like yeah, it was good. It made me accountable (Ch05’s mother, age 11, medulloblastoma, research facility sessions).

#### Program logistics

Parents and children discussed their preferred frequency, duration, location and format for the exercise program. Delivering exercise sessions in home and community-based facilities were highly valued by families. Home and community locations were reportedly more convenient, more comfortable and required less travel time than travelling to the research facility.

I definitely enjoyed it at my home. I definitely want a more, probably private area (Ch01, age 14, brain stem glioma, home sessions).

If we had to keep going back to a [research facility] or somewhere like that, it would have been almost impossible because we've got other kids and all the rest of it, we had to manage. (Ch02’s father, age 10, cerebellar astrocytoma, home sessions).

Look, I thought the home visits were amazing because it meant that you could work in with our schedules. I mean I'm a single mum.... So it's nice to know that they were able to make those home visits. So you could commit to helping out but then you were also able to meet in the middle and come and see us as well which was brilliant. (Ch06’s mother, age 11, cerebellar astrocytoma, home sessions).

Health care centres (e.g., hospitals and research facilities) were less popular with parents and children. These settings reminded families of negative experiences they had during acute treatment, and they preferred alternative settings.

Anything that's away from the hospital is good for these kids. But going into the community I think is even better. I think getting them involved in something that's community-based would be a positive thing (Ch05’s mother, age 11, medulloblastoma, research facility sessions).

All parents and children reported that 1 h was an appropriate length of time for in-person exercise sessions. Most child participants reported being tired by the end of the 1-h sessions but would not have changed the length of the session.

I'd say it was a good amount of time... I was pretty tired at the end of the sessions (Ch02, age 10, cerebellar astrocytoma, home sessions).

Well I think it was an hour, was it or 45 minutes or something but I never thought it was too long. It went really quickly and it was good (Ch04’s mother, age 10, cerebellar astrocytoma, mixed home and research facility sessions).

Most parents and children reported the 30-min thrice weekly home program to be appropriate and beneficial. Some parents found it challenging to complete all three sessions during school terms, where co- and extra-curricular activities had to be balanced with the additional commitment of the exercise program. Parents did not suggest a preferred alternative for the home exercise program and generally recognised the importance of the thrice weekly frequency.

I think it was the right amount, yeah. I don’t think you would have seen the gains if it was less but I don’t think we could have coped with doing any more (Ch04’s mother, age 10, cerebellar astrocytoma, mixed home and research facility sessions).

Parents had diverse opinions as to whether 12-weeks was an ideal length of time for an exercise program. While some thought 12 weeks was ideal, others reported the program being too long with a suggestion to align with 10-week school terms. Similar to the comments made about the frequency of the home program, parents understood that the 12-week program duration was needed to see improvements.

I think generally most things with kids probably work better in the 10-week blocks in between holidays so they don’t do it in the holidays. Like they have their time off like everybody else (Ch01’s mother, age 14, brain stem glioma, home sessions).

Parents and children were also divided as to whether they preferred the exercise program closer to the conclusion of medical treatment or further into survivorship. Some parents thought that the PACTS exercise program would be ideally placed shortly after the conclusion of hospital-based rehabilitation as an opportunity for perpetuating functional gains and progressing to more advanced skills and activities. Other parents commented that they would not have been ready to take on the commitment, and some parents felt their child was more engaged in the program further away from treatment.

Further because we would not have been ready for this until probably three years post-op, I think (Ch02’s father, age 10, cerebellar astrocytoma, home sessions).

I did wish that we had have had something like this after she got – after she’d finished the initial rehabilitation and physiotherapy, if she had something like this a little bit closer after surgery, I think it would have been very beneficial … and especially because we were knocked back for the NDIS [National Disability Insurance Scheme] and we weren’t in a position to pay for a lot of physiotherapy. We got a few free sessions but that was all. So, something like this, I think would be really beneficial (Ch04’s mother, age 10, cerebellar astrocytoma, mixed home and research facility sessions).

When parents and children were asked whether they would prefer a group-based exercise program, no consensus was reached. Some parents and children thought one-on-one sessions provided an opportunity for individualised therapy and children were more focused. Conversely, other parents reported liking the social aspects of group exercise programs but suggested a combination to one-on-one and group sessions would be preferred over group session-only programs.

I just liked also the one-on-one, not a big group. That was very nice compared to what I had before (Ch01, age 14, brain stem glioma, home sessions).

Because we did other things before that, and it was group. We tried a lot of different things, and kind of gave up, really, because we just didn’t find it that beneficial. Because I think that one-on-one and seeing him and focusing on him is brilliant. Whereas, like we went to a physio, and they’d go, okay, go home and do that for 10 weeks (Ch01’s mother, age 14, brain stem glioma, home sessions).

Ah, yeah, I mean I'm sure I know my son, Ch03 would love something like that, he's very social … yeah, a socialising event as well as active, which he loves (Ch03’s mother, age 5, cerebellar astrocytoma, mixed home and research facility sessions).

#### Activity selection

Parents appreciated the goal-directed nature of the PACTS program and liked that activities were personalised to the child’s goals, interests and current level of function. Some parents and children reported participating in more generic exercise programs in the past and feel the individualised nature of the PACTS program promoted interest and adherence, while others thought and felt the program complemented their current sports and recreation interests.

I just like how it was personalised for me. (Ch01, age 14, brain stem glioma, home sessions).

I just think it as appropriate as they did for Ch01, to customise the activities to his goals. I thought that was one of the most appealing things about the program. That it wasn’t general (Ch01’s mother, age 14, brain stem glioma, home sessions).

I guess just the tailor-made, the goal setting and that sort of thing that [Therapist] did with him. Then actually being able to have him achieve some of those goals was really, yeah, very beautiful to see (Ch03’s mother, age 5, cerebellar astrocytoma, mixed home and research facility sessions).

The most frequent comment was that the activities were enjoyable; however, some children thought some of the activities were challenging.

They were pretty fun (Ch02’s dad, age 10, cerebellar astrocytoma, home sessions).

I think just the funness of the sessions but generally she hates exercise so [laughs] (Ch04’s mother, age 10, cerebellar astrocytoma, mixed home and research facility sessions).

I had to do this sort of obstacle course, run around and that was pretty hard (Ch02, age 10, cerebellar astrocytoma, home sessions).

He had goals such as the skipping, I think we aimed for 10 single jumps over the rope. I think that was a good one for him, as I said. Then we had the dribbling, which was great too, and that the way that it was introduced was that whole skill-building where it was started off easy … I really found that, I think that really helped him (Ch03’s mother, age 5, cerebellar astrocytoma, mixed home and research facility sessions).

Most parents and children thought the home program was fun, although parents reported it was occasionally difficult to motivate their child to complete the sessions. Parents utilised planning and rewards to help adhere to the home program.

Yes, definitely a lot of rewards. Okay, now if you do it then you can go and play out in the street with your friends. (Ch03’s mother, age 5, cerebellar astrocytoma, mixed home and research facility sessions).

It would be the same day. I would always keep it the same day for Ch05 so he would know when we had to do it (Ch05’s mother, age 11, medulloblastoma, research facility sessions).

A few children asked neighbours and siblings to co-participate in the home program activities, and this was fun and motivating for them.

I really liked it because I could do it with - sometimes my friend joined in and my brother – like once, my brother joined in and then I had lots of fun *(*Ch04, age 10, cerebellar astrocytoma, mixed home and research facility sessions).

#### Connection with the therapist

Participants reported that the therapist leading the program facilitated strong engagement. Commonly reported therapist characteristics included being energetic, encouraging and approachable, as well as a willingness to be flexible with the participant’s mood, level of attention and fatigue.

She was like fun (Ch04, age 10, cerebellar astrocytoma, mixed home and research facility sessions).

I think that she really loved [Therapist] and [Therapist] made it really fun so that didn’t worry her so much (Ch04’s mother, age 10, cerebellar astrocytoma, mixed home and research facility sessions).

So, she was very energetic. Things that kids need. Very energetic, very positive, gave lots of feedback and praise to him (Ch05’s mother, age 11, medulloblastoma, research facility sessions).

#### Technology options

Parents and children preferred in-person sessions and thought telehealth was not a good alternative. Parents thought their children would be more distracted on a digital device and would receive less appropriate feedback from the therapist. Parents thought an exercise session delivered via telehealth would be better than no program at all and thought some children from other families may find it a convenient delivery channel.

No. Just no. It's too easy for a kid to just - like you're going to have to have the parent and the kid both there in front of the screen. The kid will walk away or get distracted or pick up their phone or pick up a toy (Ch02’s dad, age 10, cerebellar astrocytoma, home sessions).

Doesn't work for Ch05... He has trouble focusing. So we didn’t do anything during COVID times when we couldn’t meet … but I'm sure other kids can. I'm sure it would work. So yeah, if it had to be done that way, then I'm sure it would work (Ch05’s mother, age 11, medulloblastoma, research facility sessions).

Parents thought supplementing the home program with an exercise app could be beneficial. The app would ideally have a library of activities to complete at home that would require minimal equipment and parental supervision.

I think you guys should have your own YouTube channel where the exercises or the trainer is doing the session and then the parents - because there will be some parents that won't be able to do it with the kids for physical limitations or time or whatever and that way the kids can actually see an adult doing the activity with them. I think that would be really good (Ch02’s dad, age 10, cerebellar astrocytoma, home sessions).

## Discussion

This study explored child and parent experiences of a 12-week goal-directed therapeutic exercise program for child survivors of PFBT. Overall, participants reported the goal-directed exercise program to be valuable and helpful for improving movement competence. Key features that enhanced the feasibility and acceptability of the program included convenient community- and home-based settings, engaging activities that were tailored to the child’s needs and preferences and face-to-face delivery by a qualified therapist. These findings are consistent with the results of studies describing the experience of children and parents in other paediatric patient groups [30, 31].

Participants perceived improvements in movement competence, including the acquisition of new skills. Children reported positive changes within the context of participating in the exercise program (e.g., improved performance of activities completed during the exercise session), while some reported improvements in other contexts (e.g., physical education at school), highlighting the potential translatability of the goal-directed approach across multiple life settings. Participants did not overtly report changes in cardiorespiratory fitness, fatigue or strength, largely reporting on improvements to skills relating to their goals. As participant goals were predominantly related to skills, participants may have been more attentive to and/or expectant of changes in these specific skills than changes in function [32]. Nevertheless, families were satisfied with the goal-directed, individualised nature of the exercise program and did not report a desire for other changes.

The goal-directed approach to exercise therapy is novel in paediatric oncology. Both children and parents valued an individualised approach to training that was responsive to their needs and interests. Goal-directed interventions are inherently designed to be meaningful to the patient and sensitive to the patient’s clinical presentation and preferences [33, 34]. Structured impairment-focused interventions (e.g., resistance training to improve muscle strength) typically do not yield positive participation outcomes [21], while goal-directed approaches demonstrate a strong potential for long-term improvements through fostering the participant’s autonomy [20, 35]. Results from this study suggest that goal-directed programs are acceptable and valued by children and their families and may be more translatable to meaningful participation outcomes.

Participating families preferred the home- and community-based locations, and notably, disliked the idea of returning to health care centres (e.g., hospitals). For those children in the post-treatment phase, returning to hospital can induce fear and anxiety, as well as being inconvenient and expensive for travel [36]. Delivering the program at or near their home significantly reduced travel time to and from the exercise program, was more comfortable for children and allowed some parents to complete other domestic duties (e.g., caring for other children). The appropriate exercise setting is therefore crucial to facilitating program adherence, enjoyment and long-term participation. To better meet the needs of paediatric survivors of PFBT and their families, therapeutic exercise programs delivered in community-based settings should be considered.

Families reported the face-to-face therapist-led sessions were essential for child engagement. Nonetheless, multiple families suggested an interactive digital application, or an app, would be an effective delivery channel for the home-based exercise program. Digital exercise applications have been shown to be feasible and acceptable among childhood cancer survivors [37, 38]; however, larger trials are needed to determine their effectiveness on health outcomes. The use of digital technology may improve the reach of therapeutic exercise services for rural and remote families, who are largely underrepresented in paediatric oncology research [39].

The features of the face-to-face program (e.g., location, activities, therapist-led) supported greater adherence; however, adherence to the home program was occasionally more challenging than the face-to-face sessions. Some parents found it difficult to fit three home program sessions in their busy schedules each week, and others to motivate their child. The approach in which the home program was conducted (heavily parent-led) was challenging for some families with time constraints, such as competing co- and extra-curricular activities and/or sibling commitments. Alternative modes of exercise (e.g., digital exercise applications) could be explored when implementing exercise programs in the future to potentially reduce the involvement of parents facilitating the home programs. Some parents utilised rewards to improve adherence, while some families found involving siblings and neighbours motivating. Sibling and peer involvement was encouraged in the program, however, not utilised often. The families who utilised sibling and friend involvement claimed it was a useful tool in maintaining adherence to the home program. Overall, children were more likely to participate when they found the activities fun, reinforcing the need for activities to be games-based and suited to the child’s interests.

This study has both strengths and limitations. Firstly, it provides an in-depth description of child and parent experiences of participating in a novel goal-directed exercise program for paediatric PFBT survivors. A unique feature of this study was utilising the child-parent dyad, assessing the perspectives of the two main stakeholders in the program and understanding the factors that helped and hindered child and parent participation in the exercise program. The study followed established guidelines and a rigorous collaborative process to data analysis [29]. A limitation of this study is the small study size. There are several reasons for the small sample size, including the relative rarity of posterior fossa brain tumours, challenges in recruiting in paediatric oncology cohorts (e.g., potential burden), and recruiting during the COVID-19 pandemic [2, 40, 41]. The posterior fossa is the most common site of paediatric brain tumours and present with similar clinical features than other brain tumour types (e.g., hemispheric tumours or retinoblastomas); therefore, it was chosen as the focus of this study to bring homogeneity to the study sample [3]. Furthermore, exploratory studies, such as this study, do not necessarily need a complete description of all aspects of a novel experience to offer new insights that contribute substantially to the development of new knowledge [23]. Other limitations of this study were the lack of representation of children under 10 years and over 14 years, low-income earning households and absence of Aboriginal and Torres Strait Islander families, potentially limiting the generalisability of these results to more diverse contexts. Further, participants agreed to partake in an exercise program; therefore, they may be more motivated to seek changes to physical performance, compared to families who opted not to participate. Some of the younger child participants provided brief and/or parent-assisted responses, which could be enhanced through alternative data collection methods for children, such as drawing and diaries [42].

In summary, participants reported the PACTS goal-directed exercise intervention to be valuable and helpful for improving movement competence, including strength, fitness and other goal-specific skills. Our findings are important to the design and delivery of goal-directed exercise interventions in paediatric cancer survivors. Consideration of the location, utilisation of individualised and gamified activities and developing a connection with the therapist were key features facilitating participation in a goal-directed exercise program. Future exercise programs should explore digital delivery modes, particularly for the home program and continue to utilise family-centred, goal-directed activity selection. By identifying program features that helped and/or hindered program adherence, feasibility and acceptability, researchers and clinicians may draw relevance for designing exercise programs in the future.
